# Spinal Hypertrophic Pachymeningitis in Antineutrophilic Cytoplasmic Antibody-Negative Vasculitis: A Case Report

**DOI:** 10.7759/cureus.39121

**Published:** 2023-05-17

**Authors:** Ghassan Al-Qaysi, Mohammad Abu-Abaa, Ali Abdulsahib, Andreas Ruppel, Sajina Prabhakaran

**Affiliations:** 1 Internal Medicine, Capital Health Regional Medical Center, Trenton, USA

**Keywords:** nasal septal perforation, granulomatosis and polyangiitis, spinal canal stenosis, spinal cord compression, hypertrophic pachymeninigitis

## Abstract

Hypertrophic pachymeningitis (HPM) is a rare but extremely debilitating disease. It is even rarer for HPM to be seen in association with antineutrophil cytoplasmic antibody (ANCA)-negative vasculitis. In this case, we are presenting HPM that was diagnosed in a 28-year-old female patient who presented with worsening back pain. Imaging revealed dural-based enhancing masses affecting the thoracic spinal cord with compression. Infectious etiologies were ruled out and a total of three biopsies failed to show any evidence of granulomatous inflammation, malignancy, or evidence of immunoglobulin G4-related disease. ANCA was negative on repeated testing. The patient was managed with repeated short courses of steroids that resulted in symptomatic control as well as radiological stability of the disease. This is an extremely rare case of atypical presentation of spinal HPM that is likely associated with granulomatous and polyangiitis without other manifestations of the disease except for nasal septal perforation. This case is a supplement to a limited body of knowledge and established cases of HPM in ANCA-negative, ANCA-associated vasculitis.

## Introduction

Hypertrophic pachymeningitis (HPM) is a rare diffused or localized dural inflammatory disease characterized by dural thickening that may affect cranial and/or spinal dura with a chronic relapsing and remitting course. It can be either primary/idiopathic or secondary [1]. The most common presentation is headache [2]. Other neurological deficits can also occur depending on the location. The most useful diagnostic test is magnetic resonance imaging (MRI) [2]. It usually shows focal or diffuse dural thickening, which is nonspecific and can also be seen in other disorders such as intracranial hypotension syndrome [2]. In addition to infections, tumors, and trauma, immune-mediated HPM can be seen in antineutrophil cytoplasmic antibody (ANCA) vasculitis, immunoglobulin G4 (IgG4)-related disease, rheumatoid arthritis, sarcoidosis, Sjögren’s syndrome, and Behçet’s disease [3]. ANCA-associated vasculitis can be seen in granulomatosis with polyangiitis (GPA), microscopic polyangiitis, and eosinophilic granulomatosis with polyangiitis. The estimated prevalence of ANCA-negative vasculitis seen in association with HPM is only 3% [4].

## Case presentation

**Figure 1:**
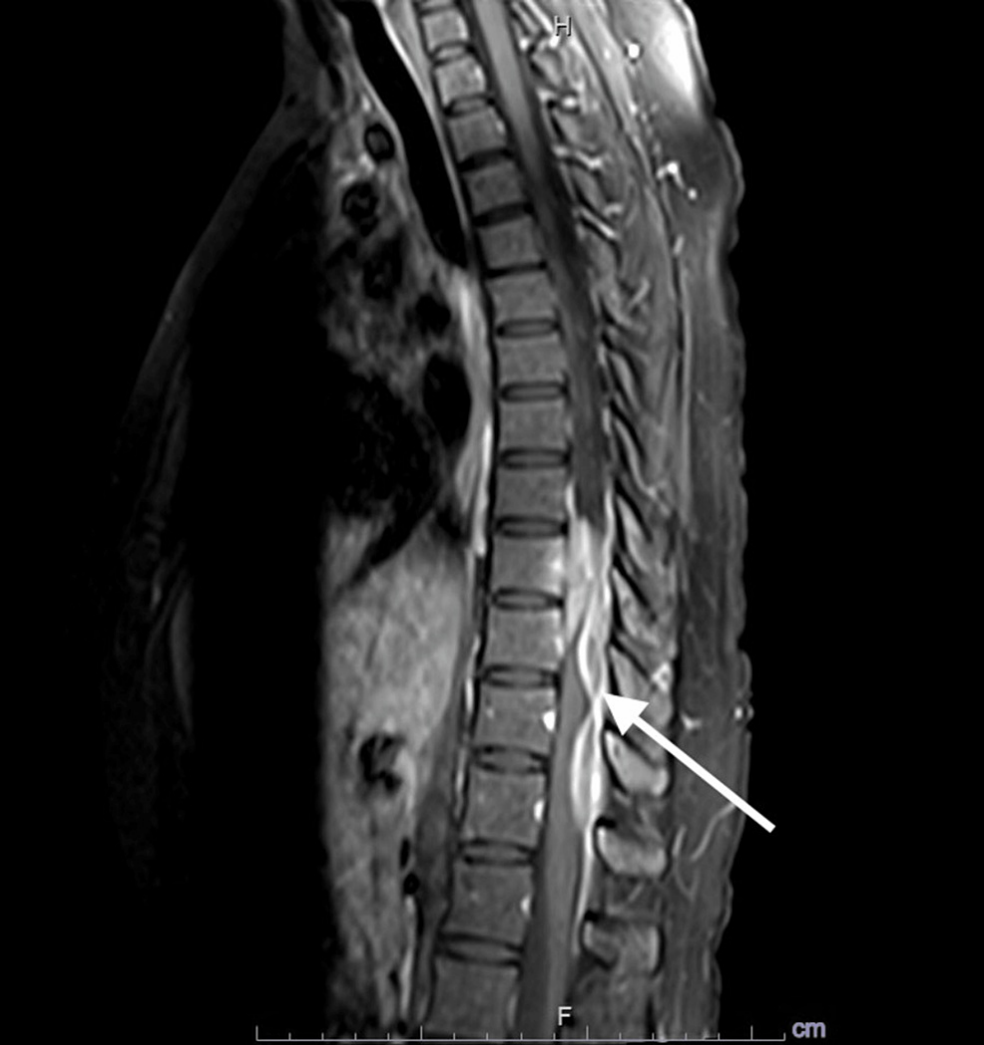
Initial magnetic resonance imaging (MRI) of the thoracic spine eight years before the current presentation. MRI of the thoracic spine showing two extramural enhancing lesions with associated spinal canal stenosis and spinal cord compression (arrow).

A 28-year-old female was referred by a neurosurgeon for evaluation of worsening symptoms. Eight years before her current presentation, she initially presented with left mid-thoracic pain that was evaluated by MRI of the spine which revealed two dural-based enhancing spinal masses at the T8 through T11 level and central canal stenosis with spinal cord compression (Figure 1). A total of three percutaneous biopsies were obtained one, four, and six years before the current presentation and sent to different tertiary centers showing only evidence of infiltrative fibroinflammatory process composed of CD3-positive/CD20-negative T cells and CD20-positive/CD3-negative B cells with a kappa-to-lambda ratio approximating 1:1.5 and no noticed evidence of granulomatous inflammation or tissue necrosis. Staining showed less than 5% of IgG4-expressing plasma cells. Pan computed tomography (CT) scan of the whole body failed to show evidence of malignancy or lymphadenopathy.

**Figure 2:**
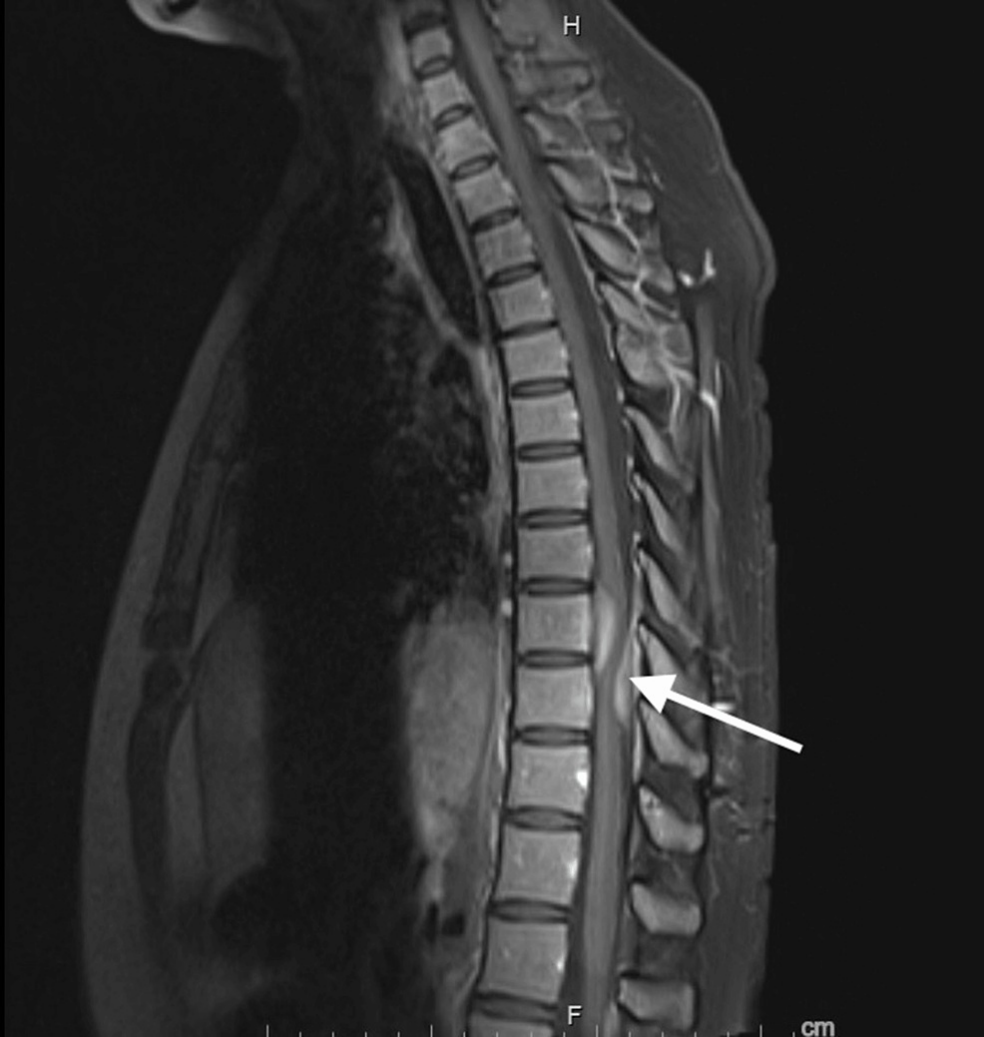
A surveillance magnetic resonance imaging (MRI) of the thoracic spine. A follow-up MRI of the thoracic spine showing relative stability/slight size improvement of the thoracic lesions coinciding with clinical improvement in response to repeated courses of steroids (arrow).

Multiple short courses of corticosteroids were pursued during these eight years, which seemed to have helped in symptom control as well as lesion regression radiologically (Figure 2). She did not have a history of tuberculosis (TB), syphilis, or Lyme disease. In addition, extensive lab investigations including ANCA were negative. Her past medical history was also remarkable for recurrent episodes of epistaxis in childhood and bilateral conductive hearing loss with tympanic membrane perforation. On this presentation, vital signs included a blood pressure of 130/90 mmHg, heart rate of 86 beats per minute, respiratory rate of 16 cycles per minute, temperature of 37.2°C, and SpO_2_ of 95% on room air. Physical examination showed diminished light touch sensation on both legs with a muscle power of 4-/5 bilaterally but did not show evidence of synovitis, nasal deformity, or sinus tenderness. Otherwise, the examination was unremarkable. Initial lab work included elevated C-reactive protein at 5.4 mg/dL; elevated erythrocyte sedimentation rate (ESR) at 50 mm/hour; normal renal function with elevated alkaline phosphatase to 150 U/L; aspartate transaminase at 32 U/L; alanine transaminase at 41 U/L; C3, C4, and IgG/IgA within normal limits and IgM slightly elevated at 15; negative antithyroid peroxidase, anti-cyclic citrullinated peptide (CCP), rheumatoid factor, anti-double-stranded DNA, anti-Smith, anti-ribonucleoprotein, and SSA/SSB antibodies; and antinuclear antibody titer of 1:640 with a homogenous pattern, 1:80 with a speckled pattern. Extensive blood work was unrevealing including negative C-ANCA, P-ANCA, and normal IgG4 level. In addition, ferritin was 73 µg/dL, gamma-glutamyl transpeptidase was elevated at 104 IU/L, and negative hepatitis C virus and human Immunodeficiency virus. Interferon-gamma release assay was also negative, and a chest X-ray did not show respiratory symptoms. Both CT and MRI of the brain were unremarkable except for nasal septal perforation.

**Figure 3:**
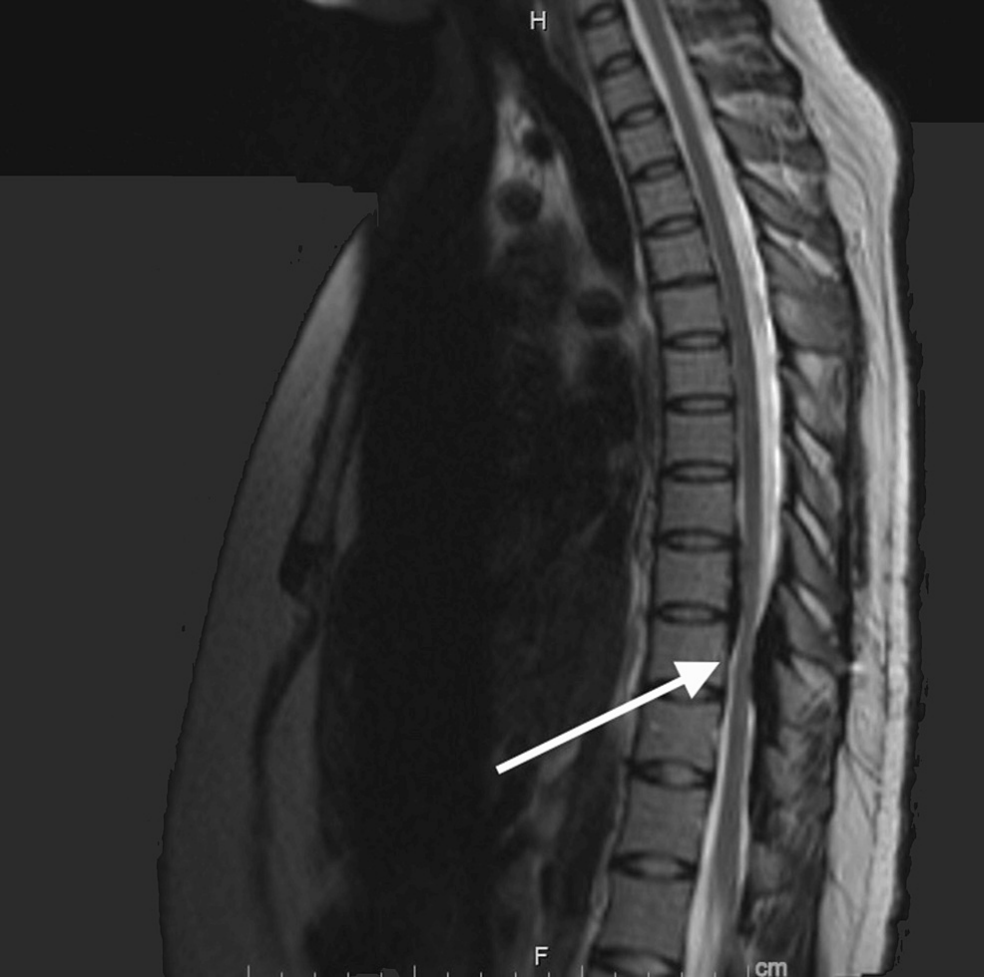
A follow-up magnetic resonance imaging (MRI) of the thoracic spine. A follow-up MRI of the thoracic spine showing relative stability in the size of spinal lesions while receiving rituximab (arrow).

Initial differential diagnosis of this pachymeningeal fibrosis included GPA, IgG4-related disease, and sarcoidosis. The patient was started on rituximab and steroids. She received three courses of six-monthly rituximab rheumatoid arthritis dosage protocol that resulted in clinical improvement of lower extremity weakness to 5-/5 with stable extradural lesions in size on serial MRI of the thoracic spine (Figure 3).

## Discussion

The first reported case of HPM dates back to 1993 [1]. HPM is a chronic inflammatory and fibrosing disease involving cranial and/or spinal dura mater. The reported prevalence of HPM is 0.949/100,000 individuals [3]. A recent retrospective study in China involving 48 patients with HPM found that the median age is 50 years [2]. Unlike prior studies showing higher prevalence in males, this study showed no sex difference in the disease distribution.

Although initial reports linked HPM to infectious processes including TB and syphilis, the exact etiology remains unclear but is believed to be an autoimmune process [5]. In addition to TB and syphilis, Epstein-Barr virus, Lyme disease, and cysticercosis have also been implicated [6]. Other etiologies include trauma, vascular abnormalities, and neoplasms [7]. However, the most common etiology of HPM according to a recent Chinese study is idiopathic HPM (67%), followed by ANCA-associated vasculitis (15%), tuberculosis meningitis (8%), viral meningitis (6%), and bacterial meningitis (4%) [2]. However, in Japan, HPM secondary to IgG4-related disease had a prevalence of 8.8% [3]. Most cases of ANCA-vasculitis associated with HPM are GPA [8]. A very recent retrospective Japanese study involving 663 patients with HPM found that GPA was serological and clinically evident in 50% of all cases of HPM and the incidence of HPM in ANCA vasculitis was 4.5%. Similar to our patient, in patients with GPA-related HPM, the manifestations were predominantly involving the ear, nose, and throat and presented with conductive hearing loss and visual disturbance [4]. The estimated prevalence of ANCA-negative vasculitis seen in association with HPM is only 3% [4].

In this case, the initial differential diagnoses of HPM included GPA, IgG4-related disease, and sarcoidosis. Nasal septal perforation can be seen in all of these diseases. Infectious etiologies, especially TB, were largely ruled out. The lack of other manifestations of IgG4-related disease as well as the presence of fewer than 5% IgG4-expressing plasma cells on staining made this a less likely possibility. Lack of granulomatous inflammation on biopsy also does make sarcoidosis a less likely possibility. Therefore, we conclude that the most likely possibility in this case is GPA. Interestingly, ANCA was negative even with repeat serology years apart. The incidence of HPM in GPA is estimated at 0.6-8% [9]. ANCA negativity has, in fact, been reported as a marker of central nervous system (CNS) involvement in GPA as 83% of ANCA-negative GPA cases have CNS involvement, while only 10% of all cases of GPA have CNS involvement [10].

Clinical presentation is variable and can include headache, progressive cranial nerve palsies, cervicodynia, and cerebellar dysfunction [7,11]. In general, it should be suspected in male patients who present with unexplained headaches or cranial nerve palsies. In HPM, headache has no specific characteristic or location, is usually progressive in nature, and is alleviated by regular analgesics [12]. A case series study of 12 cases of HPM showed that the most common presentations of HPM in decreasing order of frequency are headache, visual loss, diplopia, papilledema, other cranial nerve lesions, ataxia, and, least commonly, seizure [13]. The most commonly reported location in all cases of HPM was the sphenoid wing [13]. However, the most common location in idiopathic and TB HPM is the posterior fossa, followed by the falx cerebri, anterior fossa, cerebellar tentorium, frontal lobe, and cavernous sinuses [2].

The diagnosis is suspected based on imaging tests. These typically include CT and MRI. These usually show thickened hyperdense lesions with contrast enhancement on CT scans and hypointense lesions with contrast enhancement on MRI [14]. However, MRI and CT can be negative, leading usually to the diagnosis of a headache of unknown etiology [12]. Brain edema can also be seen [15]. A dural biopsy is considered the gold-standard diagnostic test. Typical biopsy findings include mixed inflammatory infiltrates with abundant lymphoplasmacytic cells, fibroplasia, and focal hyaline degeneration [16]. However, dural biopsy, cerebrospinal fluid analysis, and inflammatory markers such as ESR can be negative [13].

Although there is no consensus regarding the treatment of HPM, the most effective treatment is corticosteroids, with a response rate of 80% [13]. There are no guidelines regarding the dose or duration of steroid therapy in HPM. Other immunosuppressive drugs such as azathioprine and methotrexate have also been used in steroid-refractory or steroid-dependent cases [13]. Similar to our case, rituximab has been used with success in the management of HPM [13]. There is also a risk of relapse even after surgical intervention, with a time interval that ranges from weeks to years [17].

## Conclusions

HPM is a rare dural inflammatory and fibrosing disease. It most commonly presents with a headache or other neurological deficits. The majority of HPM cases are either idiopathic or associated with ANCA vasculitis. It is extremely rare for HPM to be seen with ANCA-negative vasculitis. When suspected, imaging including CT and MRI is essential to make the diagnosis. If uncertain, a biopsy should be pursued. The most commonly recommended first-line therapeutic approach is corticosteroids.
